# The Impact of Malnutrition on Acute Muscle Wasting in Frail Older Hospitalized Patients

**DOI:** 10.3390/nu12051387

**Published:** 2020-05-12

**Authors:** Maryam Pourhassan, Nikola Rommersbach, Gero Lueg, Christiane Klimek, Mirja Schnatmann, Dieter Liermann, Gregor Janssen, Rainer Wirth

**Affiliations:** 1 Department of Geriatric Medicine, Marien Hospital Herne, Ruhr-Universität Bochum, 44625 Bochum, Germany; nikola.rommersbach@posteo.de (N.R.); Gero.Lueg@elisabethgruppe.de (G.L.); Christiane.Klimek@elisabethgruppe.de (C.K.); Gregor.Janssen@elisabethgruppe.de (G.J.); Rainer.Wirth@elisabethgruppe.de (R.W.); 2 Department of Radiology, Marien Hospital Herne, Ruhr-Universität Bochum, 44625 Bochum, Germany; Mirja.Schnatmann@elisabethgruppe.de (M.S.); Dieter.Liermann@elisabethgruppe.de (D.L.)

**Keywords:** muscle mass, muscle strength, malnutrition, GLIM criteria, geriatric hospitalized patients

## Abstract

Very little is known about the effect of malnutrition on short-term changes of body composition, particularly muscle, among older hospitalized patients. We sought to investigate the association of malnutrition as assessed by the Global Leadership Initiative on Malnutrition (GLIM) criteria with changes of thigh muscle mass and muscle strength among older patients during hospitalization. Forty-one patients (age range 66–97 years, 73% female) participated in this prospective longitudinal observational study. Nutritional status was evaluated using the GLIM criteria on admission and at discharge. Functional status and mid-thigh magnetic resonance imaging (MRI) measurements of cross-sectional area (CSA) were conducted on admission and before discharge. In all, 17% were malnourished and 83% had no malnutrition. Mean mid-thigh muscle CSA declined by 7.0 cm^2^ (−9%) in malnourished patients during hospitalization (*p* = 0.008) and remained unchanged among non-malnourished patients (−1%, *p* = 0.390). Mean mid-thigh CSA of subcutaneous and intermuscular fat did not change significantly during hospitalization in both groups. Malnourished subjects lost 10% of handgrip strength (−1.8 kg) and 12% of knee extension strength (−1.5 kg) during hospitalization. However, the magnitude of both changes did not differ between groups. In a stepwise multiple regression analysis, malnutrition and changes in body weight during hospitalization were the major independent risk factors for the reduction of muscle CSA. Malnutrition according to the GLIM criteria was significantly and independently associated with acute muscle wasting in frail older patients during 2-week hospitalization.

## 1. Introduction

Sarcopenia, which is a consequence of a progressive loss of muscle mass and strength, is common among older adults and contributes to functional decline, increased risk of falls and fractures, and higher morbidity and mortality [1,2]. On average, older individuals experience annual losses of muscle mass and muscle strength of 1% and 3%, respectively [1,3]. Apart from physiological age-associated changes of body composition, other health compromising factors such as acute and chronic disease, disease-related immobilization and malnutrition may further accelerate the development of sarcopenia [4,5].

Immobilization and unintentional bed rest are probably the most important risk factors of hospitalization leading to a reduction of activities of daily living, loss of muscle mass and strength, and increased mortality [6,7]. Results of a prospective study among geriatric inpatients (mean age 82.8 ± 5.9 years) demonstrated a significant association between sarcopenia and 1-year mortality among patients with reduced mobility [8]. In addition, a previous bed rest study among 10 healthy older adults (mean age 67.0 ± 5.0 years) reported 1 kg (6%) muscle mass loss from the lower extremities after 10 days [9]. 

Besides immobility, other factors such as malnutrition may further aggravate the loss of muscle mass and muscle strength in older adults [10]. Co-occurrence of malnutrition and physical inactivity, which are both prevalent among older individuals, may speed the process, leading to sarcopenia in conjunction with serious adverse health outcomes [11,12]. In an observational, prospective cohort of 378 older hospitalized patients aged ≥70 years, Pierik et al. demonstrated a significant association between being at high risk of malnutrition and lower muscle mass on admission [4]. These findings are remarkable in the way one could hypothesize that older individuals with malnutrition would lose more muscle mass during bed rest than those with normal nutritional status. However, it remains unclear to what extent malnutrition is associated with a reduction of muscle mass during hospital stay among frail, older hospitalized patients.

A new and global definition of malnutrition has recently been proposed by the Global Leadership Initiative on Malnutrition (GLIM) [13]. In clinical settings, at least one phenotypic criterion (unintentional weight loss, low body mass index and/or reduced muscle mass) and one etiologic criterion (reduced food intake or malabsorption or inflammation) is required for the diagnosis of malnutrition based on the GLIM criteria. Accordingly, the purpose of the present study was to investigate the association of malnutrition as assessed by GLIM criteria with changes of thigh muscle mass and muscle strength among frail older patients during hospitalization.

## 2. Materials and Methods

This prospective, longitudinal observational study was undertaken between September 2017 and November 2018 at the university hospital Marien Hospital Herne in Germany. This study is an additional analysis of previously obtained data in 41 older patients (mean age 82.4 ± 6.6 years; 73% females) investigating the effect of immobility on muscle changes during hospitalization among acutely ill older adults. Therefore, patients were recruited based on their mobility status at hospital admission from the geriatric hospital department (*n* = 22) and the geriatric day clinic (*n* = 19). In the current study, we analyzed the same participants of previously obtained data.

Eligibility criteria were age ≥65 years, a probable stay of at least 14 days in hospital, ability to understand and cooperate and written informed consent. Participants with immobility for more than 3 weeks prior to admission, edema, leg amputation, heart failure, expected change in diuretic dose during hospital stay and metallic implants were excluded from the study. 

Nutritional and geriatric assessment was performed within 24 h after hospital admission (T0, baseline) except the Barthel Index, which was evaluated on admission and at the time of discharge (T1, follow-up). Muscle strength, body weight and mid-thigh magnetic resonance imaging (MRI) measurements were conducted within 24 h after hospital admission and before discharge, at the latest after 14 days. The study protocol was approved by the ethical committee of Ruhr-University Bochum (17-6048, approved on 8 August 2017).

### 2.1. Assessment of the Nutritional Status

In order to investigate the association of malnutrition with changes of muscle mass during hospitalization, patients were stratified according to their nutritional status, primarily with the Mini Nutritional Assessment Short Form (MNA-SF) [14]. After publication of the new diagnostic criteria of the Global Leadership Initiative on Malnutrition (GLIM) [13], these criteria were also applied to all study participants.

#### 2.1.1. GLIM Criteria

As phenotypic components, we evaluated unintentional weight loss >5% in the last six months and BMI below 22 kg/m^2^ if >70 years and below 20 kg/m^2^ if <70 years. Out of 41 patients, two were at age 66 and 69 years in which both had normal BMI. In addition, muscle mass (MM) was not considered as one of the phenotypic components due to unreliable quality of MM data as measured by bioelectrical impedance analysis (BIA) in our study. Reduced food intake (according to MNA-SF) and acute or chronic inflammation (CRP >2 mg/dL) were assessed as etiologic components. None of the subjects suffered from chronic gastrointestinal disease with malassimilation or malabsorption. Accordingly, patients were classified as malnourished if at least one phenotypic and one etiologic criterion was present. 

#### 2.1.2. MNA-SF

All patients were screened using the MNA-SF and patients were categorized as having normal nutritional status (12–14 points), at risk of malnutrition (8–11 points) or malnourished (0–7 points).

Both malnourished and non-malnourished patients received similar nutrition, except an oral nutrition supplement was provided for patients with MNA-SF <8. In addition, both groups had physical training for at least 30 min twice a day as a routine rehabilitation program. However, immobile patients who were more or less bedridden participated less. Furthermore, an individualized training program was provided to all patients according to the deficient in activity of daily living.

### 2.2. Geriatric Assessment 

Activities of daily living were determined using the Barthel Index [15]. The point range of the German version of the Barthel Index is 0–100 pts., with 100 pts. indicating independency in all activities of daily living. Mobility status was defined according to walking ability as described by the respective item of the Barthel Index. Briefly, a mobility score of either 15 (can sit or walk at least 50 m independently without a walker or help but may use any aid i.e., stick) or 10 (can walk at least 50 m with a walker or with help of one person) was considered to be mobile. Participants with a mobility score of 5 (walking the distances in the living area with help; wheelchair independent) and 0 (patients do not meet these criteria, immobile) was considered to be immobile. The FRAIL scale [16] was used to identify persons at risk of frailty; a score of 0 is considered not frail whereas scores of 1–2 and 3–5 are considered as pre-frail and frail, respectively. The risk of sarcopenia was investigated with the use of SARC-F questionnaire [17], which ranges from 0 to 10 and subjects with score ≥4 were defined as having probable sarcopenia. Furthermore, medical comorbidities were evaluated using the Charlson Comorbidity Index (CCI) [18].

### 2.3. Assessment of Muscle Strength

Isometric knee extension strength (Strength measuring device FK, Sauter GmbH, Balingen, Germany) was measured according to the protocol described by Gandevia [19]. Briefly, knee strength was assessed with the patient in a seated position with a strap around the leg 10 cm above the ankle joint with the hip and knee joint angles positioned at 90 degrees. Handgrip strength (HGS) was assessed using a Jamar dynamometer (Lafayette Instrument Company, Lafayette, IN, USA). Handgrip and knee strengths were measured three times at the dominant or unaffected side of hand/leg and the maximum score was recorded.

### 2.4. Mid-Thigh Magnetic Resonance Imaging (MRI) Cross Sectional Area

**Figure 1:**
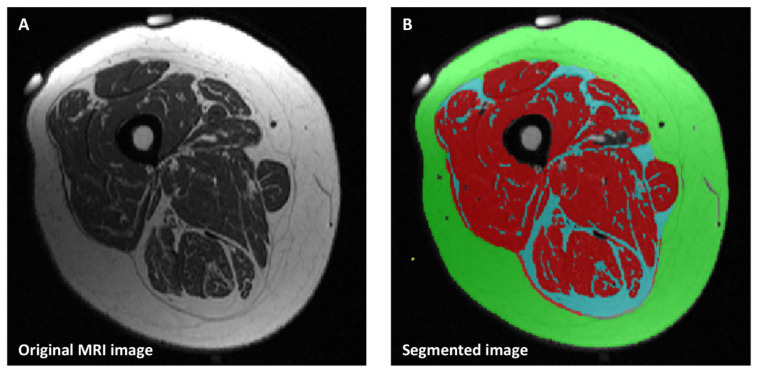
A sample of single-slice mid-thigh MRI image before (**A**) and after (**B**) segmentation. Structures in red: muscle, green: subcutaneous fat, blue: intermuscular fat.

Mid-thigh muscle, subcutaneous and intermuscular fat cross-sectional area (CSA) were measured using MRI scans (Siemens Magnetom Sonata, 1,5 Tesla, Erlangen, Germany). Briefly, the middle length of the non-affected, preferably dominant thigh was determined and marked with a semi-circumferential line drawn with a permanent marker for replication at the time of follow-up. Directly before the MRI measurement, two MRI-detectable capsules were fixed at both ends of the line (Figure 1A). Five T1-weighted transversal scans with a slice thickness of 4 mm were obtained and the single slice with both landmarks was selected for segmentation. The following imaging parameters were used: 447 ms repetition time (TR), 13 ms echo time (TE), 160° flip angle (FA) and 400 × 300 mm^2^ field of view (FOV) with a resolution of 1.2 × 0.8 mm^2^. The field of view and the settings were kept constant throughout all measurements. Single slice CSA of muscle, subcutaneous and intermuscular fat at mid-thigh were manually segmented (Figure 1B) by using the SliceOmatic software (version 5.0; Tomovision, Montreal, Canada). The segmentation of MRI images was blinded for subjects’ characteristics.

### 2.5. Statistical Analysis

The statistical analysis was performed using SPSS statistical software (SPSS Statistics for Windows, IBM Corp, Version 26.0, Armonk, NY, USA). With the expected 0% change in muscle mass of mobile patients and 2.7% loss of muscle mass in immobile patients with a realistic high standard deviation of ± 3%, a case number of *N* = 40 in a 1:1 design with a power of 0.8 and a Type I error of 0.05 was calculated (http://PowerAndSampleSize.com). Continuous variables are reported by means and standard deviations (SDs) for normally distributed variables and median values with interquartile ranges (IQR) for non-normally distributed data. Categorical variables are shown as *n* (%). Comparison of variables at baseline and magnitude of changes at follow-up between the categories of MNA-SF were analyzed by using a one-way ANOVA with post-hoc Tukey and Kruskal–Wallis H test for normally and non-normally distributed variables, respectively. In order to compare the nutritional groups according to GLIM criteria, we performed an unpaired samples *t* test and Mann–Whitney *U* test for parametric and nonparametric data, respectively. 

Differences in variables between baseline and follow-up within each nutritional group were analyzed by using paired samples *t* test and Wilcoxon signed rank for normally and non-normally distributed values, respectively. Categorical variables were compared by the Chi square test. In addition, a stepwise multiple regression analysis was performed to investigate the independent effects of age, gender, body weight changes during hospital stay, weight loss in 6 months, nutritional status, mobility, inflammation and disease on reduction of muscle mass as dependent variable. A *p*-value of <0.05 was considered as the limit of significance.

## 3. Results

### 3.1. Characterization of Study Population

Table 1 shows the characteristics of the study population at baseline stratified by nutritional status according to GLIM criteria. Major diagnoses defined as reason for hospital admission were falls and fractures, pneumonia, osteoarthritis, post-stroke care and urinary tract infection. Of the total population with age range 66–97 years, 17% were malnourished and 83% had no malnutrition according to GLIM criteria. Further details concerning the prevalence of malnutrition according to MNA-SF can be obtained from Table 1 and Table 2. 

All patients in the malnourished group were immobile and had significantly more unintentional weight loss in the last six months than the non-malnourished group. Malnourished patients displayed more frailty and had lower total MNA-SF, Barthel Index and mobility compared to those without malnutrition. Both malnourished and non-malnourished patients received similar nutrition except for oral nutrition supplement which was only provided for patients with MNA-SF < 8. In addition, both groups had physical training for at least 30 min twice a day as a routine rehabilitation program. However, immobile patients who were more or less bedridden participated less.

There were no statistically significant differences either in average length of stay (*p* = 0.577) or in time between baseline and follow-up MRI scans (*p* = 0.667) between malnourished and non-malnourished patients. 

### 3.2. Changes of MRI-CSA during Follow-Up 

Detailed results of mid-thigh CSA obtained by MRI of the study participants stratified by nutritional status are given in Table 2. 

#### 3.2.1. GLIM Criteria

At baseline, there were no significant differences in mean mid-thigh CSA of muscle and intermuscular fat between malnourished and non-malnourished groups, whereas mean mid-thigh CSA of subcutaneous fat was significantly lower (*p* = 0.047) in malnourished patients.

**Figure 2:**
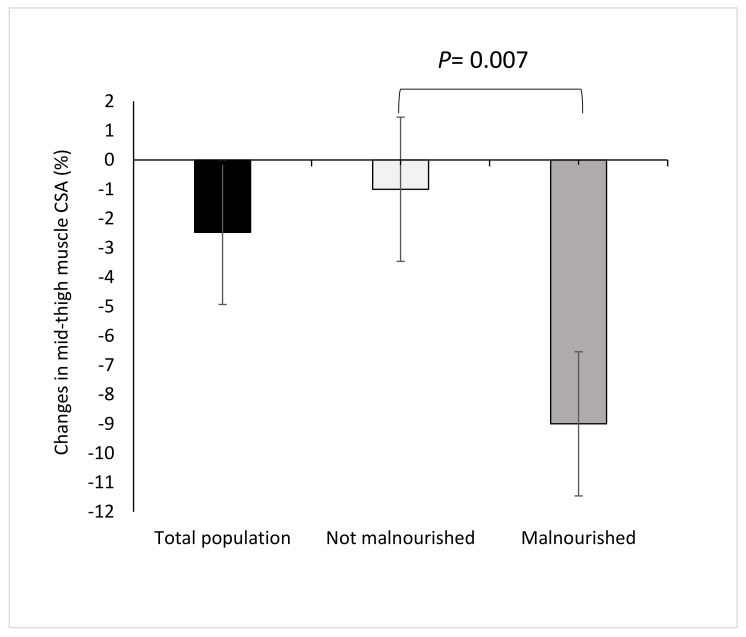
Changes in mean mid-thigh muscle cross sectional area (CSA) as a percentage of initial muscle area during hospitalization in total population (*n* = 41), in malnourished (*n* = 7) and non-malnourished (*n* = 34) patients according to Global Leadership Initiative on Malnutrition (GLIM) criteria.

In the total population, there was a significant decline in absolute mean mid-thigh muscle CSA during hospitalization by 1.8 cm^2^ (*p* = 0.043). In malnourished patients, mean mid-thigh muscle CSA significantly declined by 7.0 cm^2^ from baseline to follow-up (*p* = 0.008) and remained unchanged among the non-malnourished patient group (*p* = 0.390, Table 2). The magnitude of reduction of mean mid-thigh muscle CSA as a percentage of initial muscle mass was significantly higher in the malnourished compared to non-malnourished patients (−9% vs. −1%, respectively; *p* = 0.007, Figure 2). Mean mid-thigh CSA of MRI of subcutaneous and intermuscular fat did not change significantly during hospitalization in both groups. 

#### 3.2.2. MNA-SF

At baseline, there were no significant differences in mean mid-thigh CSA of muscle and subcutaneous fat between the categories of MNA-SF, whereas mean mid-thigh CSA of intermuscular fat was significantly lower (*p* = 0.002) in malnourished patients. 

Mean mid-thigh muscle CSA significantly decreased by 6.0 cm^2^ during hospitalization in malnourished patients (*p* = 0.036) and remained unchanged over time in patients at risk of malnutrition (*p* = 0.166) and those with normal nutritional status (*p* = 0.771). The magnitude of decline of mean mid-thigh muscle CSA as a percentage of initial muscle mass was significantly higher in the malnourished patients than the patients with normal nutritional status (−8% vs. −0.4% *p* = 0.045). No significant changes of mean mid-thigh CSA of subcutaneous and intermuscular fat could be detected across the categories of MNA-SF during follow-up.

### 3.3. Functional Status

Comparison of functional status and activity of daily living of study population are summarized in Table 3. Malnourished and non-malnourished groups did not significantly differ at baseline for handgrip strength. However, handgrip strength was significantly lower in malnourished than non-malnourished patients at follow-up. In addition, lower mean knee extension strength was observed in the malnourished compared to non-malnourished group on admission and at time of discharge. Malnourished subjects lost 10% of handgrip strength and 12% of knee extension strength during hospitalization. However, the magnitude of both changes did not differ significantly between groups. The Barthel Index improved in both groups overtime. Similar results using MNA-SF were observed.

In a stepwise multiple regression analysis, the effects of age, gender, body weight on admission, weight loss in last six months, changes in body weight during hospitalization, mobility, malnutrition, mid-thigh muscle mass area on admission, inflammation (CRP) and comorbidity (as independent variables) on absolute reduction of mid-thigh muscle mass area (as dependent variables) were tested (Table 4). Malnutrition as assessed by GLIM criteria followed by changes in body weight during hospital stay were the major independent risk factors for absolute reduction of mid-thigh muscle mass area during hospitalization. In addition, using malnutrition as assessed by MNA-SF in the regression model demonstrated a slightly significant effect (*p* = 0.074) in absolute reduction of mid-thigh muscle mass area.

## 4. Discussion

In many older persons, sarcopenia is the consequence of both a continuous decline of muscle mass and strength over years, which is superimposed by acute losses due to periods of disuse and disease. In this study, we aimed to investigate the association of malnutrition with changes of thigh muscle mass and muscle strength. The major finding of this prospective longitudinal study is that malnutrition according to the GLIM criteria and weight loss were the only independent risk factors for a significant decrease of mid-thigh muscle CSA during two weeks of hospitalization in frail older persons. Notably, malnourished subjects based on the GLIM criteria, experienced an average loss of 9% of thigh muscle mass within 14 days of illness, which is comparable to the average decline of muscle mass during nine years of aging in older persons. Similarly, muscle mass significantly decreased during hospitalization in malnourished patients based on MNA-SF. We hypothesized that immobility, malnutrition, inflammation and age would be risk factors for acute muscle wasting, however we did not expect that malnutrition would be the major one.

Malnutrition, which is frequently seen in older patients, can lead to changes of body composition resulting in functional impairment and poor clinical outcomes. Beyond physiological muscle atrophy with advancing age, low muscle mass as a clinical result of malnutrition leads to functional decline, increased risk of falls and fractures, a lower quality of life and higher morbidity and mortality [20,21]. In a large cohort study of older hospitalized patients (mean age 79.7 ± 6.39 years), Verlaan et al. demonstrated a higher survival rate three months after discharge in patients with greater muscle mass and without malnutrition at hospital admission [22]. In addition, Pierik et al. reported the association of being at high risk of malnutrition with lower muscle mass at admission in the same study population [4]. However, despite the high prevalence of inactivity and malnutrition in the study by Pierik et al., the authors did not find a significant loss of muscle mass or fat free mass, as measured by BIA, in both malnourished or non-malnourished patients during five days of hospitalization [4], which seems contrary to our results. In this study, patients with malnutrition based on the GLIM criteria, experienced 9.0% loss (0.5 cm^2^ per day) in MRI-derived mid-thigh muscle CSA during the course of a 14-day hospitalization period, whereas muscle CSA did not change in patients without malnutrition. It is worth mentioning that our patients had a longer median hospital stay compared to the latter study [4] and we assessed muscle mass using MRI scans, which provide a very sensitive and accurate measurement. However, BIA can be considered as a validated tool for measuring body composition in hospital settings [23,24]; BIA measurement is highly influenced by hydration status, which may underestimate or cover muscle mass loss during hospitalization [22,25]. Furthermore, all our malnourished patients in the current study were immobile, frail and probably sarcopenic at the time of admission. The combination of these factors may have led to the detection of higher muscle mass loss in the malnourished patients in our study. 

Another finding of our study was that the activities of daily living as measured by Barthel Index were significantly lower in patients with malnutrition according to the GLIM criteria on admission and at the time of discharge, although Barthel Index improved in both groups during the hospital stay. This is supposed to be the effect of the routine rehabilitation program in our geriatric acute care unit, i.e., physical and occupational training in all patients and the use of high protein oral nutritional supplements (ONS) in malnourished patients. Remarkably, the substantial reduction of muscle mass in this study occurred despite training and nutritional therapy. We hypothesize that the rate of muscle mass and strength loss would be much higher without this support. Nutrition, and more specifically protein intake, play a central role in muscle protein synthesis and maintaining muscle mass [26]. A systematic review and meta-analysis reported that the use of high protein ONS in the clinical setting and after hospital discharge was associated with improvement in functional status and a reduction of complications [27]. In addition, results of a multicenter, randomized, controlled trial among 380 sarcopenic older adults mean age 77.7 years demonstrated improvements in muscle mass and lower-extremity function during a 13-week intervention with a high protein ONS [28].

In contrast to their non-malnourished counterparts, patients with malnutrition according to the GLIM criteria showed lower handgrip and isometric knee extension strength on admission and at the time of discharge. Our findings demonstrated an obvious tendency towards a further decrease in handgrip and isometric knee extension strength during hospital stay in malnourished patients only; however, the magnitude of changes did not reach the level of significance likely due to the small number of malnourished patients. This finding is in concordance with a previous study of malnourished older hospitalized patients (mean age 81.7 ± 5.1 years), in which malnourished subjects had lower muscle mass and tended to have lower strength than non-malnourished patients [29].

Previous studies in older adults have indicated that low muscle strength was associated with higher risk of falls [30,31]. In a prospective cohort of older patients, Van Ancum et al. found that patients with lower muscle strength and muscle mass at admission were at risk of a higher number of geriatric conditions including malnutrition, falls and functional disability [25]. It can be speculated that malnutrition in combination with physical inactivity does not only cause muscle wasting but may also have adverse impacts on muscle strength and physical functioning of the upper and lower extremity of older individuals. Our data underline the need for routine nutritional assessment and early recognition of malnutrition in older hospitalized patients. In addition, minimizing muscle mass loss and preserving muscle strength should be part of the treatment and management of malnutrition in these patients [20]. However, MRI scans of muscle will probably not be available for all geriatric patients.

This study has some limitations. We did not measure physical activity directly. Definition of mobility was according to walking ability as described by the Barthel Index, which might raise some concern. However, reliability of mobility measurement as assessed by Barthel Index was reported in stroke patients [32]. In addition, there was a shorter follow-up period of MRI scans for some patients during hospitalization, mostly due to organizational issues. However, this did not differ between both groups. It is also worth noting that our study participants comprised immobile patients from a geriatric hospital department (*n* = 22) and mobile patients from a geriatric day clinic (*n* = 19). Patients from the geriatric day hospital had better functional and nutritional status compared to those from the geriatric hospital department. Therefore, the study population demonstrated a small number of malnourished participants and a relatively low prevalence of malnutrition. Furthermore, our study had a small sample size due to difficulties recruiting acutely immobilized multi-morbid older patients. However, using the MRI technology as a gold standard allowed us to precisely assess the quantitative changes in body composition during hospitalization. Further prospective interventional research is required to determine the effect of high protein ONS and physical training on muscle mass and physical function including muscle strength. 

## 5. Conclusions

Malnutrition was significantly and independently associated with muscle wasting in frail older patients during hospitalization. These data emphasize the importance of proper nutrition e.g., high protein ONS and physical training for muscle health and should stimulate respective interventional trials in frail older patients.

## Figures and Tables

**Table 1:** Characteristic of study population at baseline (T0) stratified by nutritional status according to GLIM criteria.

		GLIM Criteria		
	All (*n* = 41)	Malnourished (*n* = 7; 17%)	Non-Malnourished (*n* = 34; 83%)	*p* Value
Gender				
Female (number, %)	30 (73)	4 (57)	26 (77)	0.361
Male (number, %)	11 (27)	3 (43)	8 (23)	
Age (y)	82.4 ± 6.6	81.1 ± 8.0	82.6 ± 6.3	0.660
Height (m)	1.61 ± 0.1	1.67 ± 0.1	1.60 ± 0.1	0.036
Actual body weight (kg)	73.9 ± 16.8	66.5 ± 13.1	75.4 ± 17.2	0.150
BMI (kg/m^2^)	28.4 ± 6.4	23.5 ± 3.8	29.4 ± 6.4	0.006
Weight loss in 6 months (kg)	2.2 ± 3.1	5.8 ± 3.0	1.4 ± 2.6	0.006
CRP (mg/dl)	2.5 ± 5.5	2.5 ± 1.9	2.5 ± 6.0	0.969
Total MNA-SF, Median (IQR)	8 (10–12)	9 (7–9)	11 (9–12)	0.010
Barthel Index on admission, Median (IQR)	55 (40–67)	35 (35–45)	60 (40–70)	0.005
Walking, Median (IQR)	5 (5–10)	5 (5–5)	10 (5–10)	0.025
Frail Simple scale score, Median (IQR)	3 (2–3)	3 (3–4)	3 (2–3)	0.006
SARC-F scores, Median (IQR)	6 (4–7)	5 (3–8)	6 (4–7)	0.747
CCI score, Median (IQR)	2 (1–3)	1 (1–3)	2 (1–2)	0.959
Mobility Status				
Mobile (number, %)	19 (46)	0 (0)	19 (56)	0.010
Immobile (number, %)	22 (54)	7 (100)	15 (44)	
Length of hospital stay	16 (14–18)	16 (14–17)	16 (14–18)	0.577
Time between MRI scans	14 (11–14)	14 (11–14)	13 (11–14)	0.667

GLIM, Global Leadership Initiative on Malnutrition; CRP, C-reactive protein; MNA-SF, Mini Nutritional Assessment Short Form (normal nutritional status 12–14 points, at risk of malnutrition 8–11 points and malnourished 0–7 points); Frail Simple scale (not frail with score 0, pre-frail with scores of 1–2 and frail with 3–5); SARC-F scores (high risk of sarcopenia with score ≥4); CCI, Charlson Comorbidity Index; MRI, Magnetic Resonance Imaging. Values are given as mean ± SD, number (%) or median (IQR, interquartile range).

**Table 2:** Comparison of mean mid-thigh MRI cross sectional area (cm^2^) of study population stratified by nutritional status at baseline (T0) and follow-up (T1) and the respective changes.

	MNA-SF All (*n* = 41)			GLIM Criteria All (*n* = 41)	
Mid-thigh MRI cross sectional area (cm^2^)	Malnourished (*n* = 5, 12%)	At risk (*n* = 22, 54%)	Normal (*n* = 14, 34%)	Malnourished (*n* = 7, 17%)	Non-malnourished (*n* = 34, 83%)
Muscle area T0	68.1 ± 15.2	82.6 ± 19.7	83.5 ± 15.9	78.9 ± 21.0	81.6 ± 17.9
Muscle area T1	62.1 ± 11.1 †	80.8 ± 21.2 ^a^	83.1 ± 16.6 ^bb^	71.9 ± 20.8 ††	80.8 ± 19.2
Changes in muscle area	−6.0 ± 4.3	−1.8 ± 6.0	−0.4 ± 5.2 ^b^	−7.0 ± 4.7	−0.7 ± 5.3 ^c^
Subcutaneous fat area T0	77.1 ± 48.0	77.6 ± 43.0	112.4 ± 64.4	63.8 ± 28.6	94.7 ± 55.8 ^c^
Subcutaneous fat area T1	70.0 ± 41.8	75.5 ± 40.9	106.6 ± 59.2	56.9 ± 27.4	91.3 ± 51.0 ^c^
Changes in subcutaneous fat area	−7.1 ± 9.4	−2.1 ± 12.0	−5.7 ± 19.7	−6.8 ± 7.8	−3.4 ± 15.8
Intermuscular fat area T0	9.4 ± 2.6	18.2 ± 8.8	21.1 ± 10.7 ^bb^	14.2 ± 6.5	18.9 ± 9.9
Intermuscular fat area T1	9.5 ± 2.6	17.3 ± 8.8	21.0 ± 13.1	12.0 ± 5.1	18.7 ± 11.0 ^c^
Changes in intermuscular fat area	0.1 ± 0.9	−0.9 ± 3.2	−0.1 ± 3.8	−2.2 ± 2.7	−0.2 ± 3.2

MRI, Magnetic Resonance Imaging; MNA-SF, Mini Nutritional Assessment Short Form; GLIM, Global Leadership Initiative on Malnutrition. ^a^ *p* < 0.05, difference between malnourished and at risk of malnutrition based on MNA-SF; ^b^ *p* <0.05, ^bb^ *p* < 0.01 difference between malnourished and normal nutritional status based on MNA-SF; ^c^ *p* < 0.05, difference between malnourished and non-malnourished based on GLIM criteria; † *p* < 0.05, †† *p* < 0.01 difference within group from T0 to T1. Values are given as mean ± SD.

**Table 3:** Comparison of functional status of study population stratified by nutritional status at baseline (T0) and follow-up (T1) and the respective changes.

	GlIM Criteria		*p* Value
Functional Status	Malnourished (*n* = 7, 17%)	Non-Malnourished (*n* = 34, 83%)	
Handgrip strength (kg), T0	17.3 ± 5.7	20.3 ± 8.7	0.268
Handgrip strength (kg), T1	15.4 ± 4.2	20.8 ± 8.8	0.024
Changes in Handgrip strength (kg)	−1.8 ± 4.3	0.5 ± 2.4	0.207
Knee extension strength (kg), T0	12.4 ± 4.6	17.5 ± 6.8	0.030
Knee extension strength (kg), T1	10.9 ± 4.7	17.2 ± 7.0	0.022
Changes in Knee extension strength (kg)	−1.5 ± 2.2	−0.3 ± 3.1	0.289
Activity of daily living			
Barthel Index, Median (IQR); T0	35 (35–45)	60 (40–70)	0.005
Barthel Index, Median (IQR); T1	55 (50–75) †	75 (65–85) †	0.028
Changes in Barthel Index, Median (IQR)	20 (10–30)	15 (10–20)	0.584

GLIM, Global Leadership Initiative on Malnutrition. † *p* < 0.01, difference within group from T0 to T1.

**Table 4:** Stepwise multiple regression analysis of risk factors associated with changes in mid-thigh muscle mass area in total population (*n* = 41).

	Beta Coefficient	SE	*p* Value
Changes in absolute mid-thigh muscle mass area			
Age	0.085	0.137	0.838
Gender	−1.506	2.861	0.747
Body weight on admission	0.150	0.074	0.325
Weight loss in last 6 months	0.012	0.299	0.110
Malnutrition based on GLIM criteria	−6.271	2.088	0.005
Changes in body weight during hospital stay	0.323	0.155	0.044
Mid-thigh muscle mass area on admission	−0.178	0.078	0.864
Mobility based on Barthel Index	0.344	0.255	0.079
Frail Simple scale score	−1.224	1.314	0.101
C-reactive protein	−0.240	0.177	0.130
Charlson Comorbidity Index	1.629	0.889	0.284

GLIM, Global Leadership Initiative on Malnutrition; SE, standard error.
